# Migration-Related Trauma Among Asylum Seekers Exposed to the Migrant Protection Protocols

**DOI:** 10.1001/jamanetworkopen.2025.50786

**Published:** 2026-01-06

**Authors:** Kyle Joyner, Elizabeth Burner, Sarah Axeen, Sandhya Murugan, Katya English, Todd W. Schneberk

**Affiliations:** 1 Department of Emergency Medicine, Keck School of Medicine, University of Southern California, Los Angeles; 2 Suzanne Dworak-Peck School of Social Work, University of Southern California, Los Angeles

## Abstract

**Importance:**

The Migrant Protection Protocols (MPP) were introduced in January 2019 and changed US asylum procedures by requiring certain asylum seekers to remain in Mexico while awaiting immigration proceedings. Understanding the association of MPP with trauma is important for informing immigration and health policy.

**Objective:**

To evaluate whether exposure to the MPP was associated with trauma reported during migration.

**Design, Setting, and Participants:**

This retrospective cohort study of forensic medical evaluations in both the US and Mexico was conducted from 2016 to 2022. The study included 94 asylum seekers who underwent forensic medical evaluations between January 1, 2016, and December 31, 2022, categorized into 3 groups: pre-2019 asylum seekers (n = 26), 2019-2022 asylum seekers exposed to the MPP (n = 22), and 2019-2022 asylum seekers not subjected to the MPP (n = 46). Statistical analysis was performed from January 2023 to May 2025.

**Exposures:**

Exposure to the MPP, a US immigration policy implemented in January 2019 that required certain asylum seekers to remain in Mexico during their immigration proceedings.

**Main Outcomes and Measures:**

The primary outcome was traumatic experiences during migration. Secondary outcomes included posttraumatic stress disorder (PTSD) based on the forensic examiner’s diagnosis and subgroup analyses by geographic region. Logistic regression was used to assess differences in reported trauma. Qualitative narratives were included to contextualize the quantitative findings.

**Results:**

Among 94 participants, the mean (SD) age was 33.1 (10.1) years, 56 (60%) were men, and 68 (72%) were from Latin America. Traumatic migration events were reported by 14 of 22 participants (64%) in the MPP group compared with 3 of 26 (12%) in the pre-2019 group (odds ratio, 9.20; 95% CI, 2.12-39.89). PTSD was observed in 21 of 22 (95%) in the MPP group compared with 11 of 26 (42%) in the pre-2019 group (odds ratio, 28.64; 95% CI, 3.33-246.25).

**Conclusions and Relevance:**

In this cohort study, exposure to MPP was associated with higher rates of trauma during migration among asylum seekers. Such policies may be associated with adverse health outcomes for asylum seekers, with potential downstream implications for US public health and security.

## Key Points

**Question:**

Was exposure to the Migrant Protection Protocols (MPP; the “Remain in Mexico” policy) associated with higher rates of trauma during migration among asylum seekers?

**Findings:**

In this cohort study of 94 asylum seekers evaluated between 2016 and 2022, 14 of 22 participants (64%) in the MPP group reported trauma during migration compared with 3 of 26 (12%) in the pre-2019 group. This result was statistically significant.

**Meaning:**

These findings suggest that restrictive border policies may have unintended consequences on migrant health, with downstream implications for US public health and security.

## Teaser

This cohort study evaluates whether exposure to the Migrant Protection Protocols was associated with trauma reported during migration among asylum seekers between 2016 and 2022.

## Introduction

Asylum seekers are individuals who apply for international protection due to fears of persecution or violence in their home countries.^1^ In 2024, the United Nations estimated that 123.2 million people were forcibly displaced worldwide due to persecution, conflict, violence, human rights violations, or other events disturbing public order.^2^ Among these displaced individuals, many sought asylum in countries with established protective programs, such as the US, where pending asylum cases exceeded 2.2 million in 2025.^3^

This unprecedented increase in asylum cases has coincided with increasingly restrictive US border policies. Citing border security concerns, the US implemented the Migrant Protection Protocols (MPP) in January 2019, requiring asylum seekers at the US-Mexico border to remain in Mexico while awaiting hearings. The MPP marked a shift from international norms, which traditionally permitted asylum seekers residence in the host country during case processing. Individuals subjected to the MPP reported being returned to border towns where they faced dangers including threats of violence and kidnapping, forced family separation, and unstable living conditions, including living in overcrowded tent cities without access to running water or electricity.^4^ Objective systematic data on trauma in this population are limited, although nongovernmental organizations such as Human Rights First and Amnesty International estimate the number of violent attacks on individuals subjected to the MPP to be over 13 000, with the true number likely higher due to underreporting.^5,6^

Due to these concerns, the MPP remained subject to legal and policy challenges and was suspended temporarily in January 2021, formally halted after a Supreme Court ruling in June 2022, and reinstated again by the US Department of Homeland Security in January 2025.^7^ In parallel, other policies such as Title 42 expulsions based on public health safety with the COVID-19 pandemic and subsequent asylum transit bans limiting asylum applications for individuals who had already traversed multiple countries similarly restricted access at the US southern border, showing a broader transition in the US asylum landscape toward more restrictive migration policies.^8,9^

Such measures not only limited access to asylum but also placed asylum seekers, a group already fleeing persecution and violence in their home countries, into unstable environments associated with a heightened risk of trauma. Global research has consistently documented high rates of trauma-related conditions among asylum seekers, including a meta-analysis of more than 5000 adults in 2020 that estimated a posttraumatic stress disorder (PTSD) prevalence of 31%.^10,11^ A more recent 2021 study of 57 asylum seekers from Central America, a region impacted by the MPP, showed PTSD rates approaching 79%.^12^ With respect to the MPP, several small case series and reports from nongovernmental organizations have raised concerns about the exacerbation of trauma for those subjected to the MPP.^13,14,15,16,17^ However, larger systematic studies on individuals exposed to the MPP remain limited, in part due to challenges with access and follow-up in this displaced population. To address this gap, forensic medical evaluations and legal affidavits, which are frequently collected to support asylum claims by providing objective documentation of trauma, could serve as valuable resources for examining the association of health outcomes with the MPP. To better understand the association of exposure to the MPP with migration-related trauma, we examined legal affidavits and forensic medical examinations of 94 asylum seekers at an academic-affiliated asylum clinic, comparing traumatic experiences before and after the implementation of the MPP in 2019.

## Methods

This retrospective cohort study evaluated traumatic experiences before and after the introduction of the MPP in January 2019. Participants were selected between January 1, 2016, and December 31, 2022, from an academic-affiliated asylum clinic providing pro bono forensic evaluations to asylum seekers. These forensic medical evaluations were performed for asylum seekers to document objective findings of their previous trauma and are associated with a higher rate of asylum application success. A study of over 2800 asylum applicants between 2008 and 2018 found that nearly 82% of those applying for immigration relief were granted relief compared with only 42% without a forensic medical examination.^18^ Clinicians performing the forensic medical evaluations undergo training to follow the Istanbul Protocol, which is a set of international guidelines for the effective investigation and documentation of torture and other human rights violations.^19^ Evaluations were conducted by the University of Southern California Human Rights Clinic in Los Angeles, California, and Tijuana, Mexico, as well as remotely due to limitations with the COVID-19 pandemic. Eligibility criteria included all asylum seekers aged 18 years or older who completed forensic medical examinations at the clinic during this period. Exclusions included age younger than 18 years, examinations missing substantial data, and cases containing potentially identifiable material even after deidentification. Of 108 forensic evaluations screened, 14 were excluded (9 individuals younger than 18 years, 3 records with duplicate information, and 2 with insufficient data) leaving 94 cases for analysis (eFigure in Supplement 1). Data from legal declarations and forensic exams were securely stored in REDCap and deidentified. Ethical approval was obtained from the University of Southern California Social Behavioral Institutional Review Board, which waived the requirement of informed consent due to the research being retrospective and posing minimal risk to participants with deidentified data. The study followed the Strengthening the Reporting of Observational Studies in Epidemiology (STROBE) reporting guideline for cohort studies.

A standardized data abstraction form was developed to record demographic characteristics (age, sex, and country of origin) and study outcomes focusing on MPP exposure, traumatic events, and reporting of PTSD. Exposure to the MPP was classified from the legal documentation and corroborated by forensic examiner report. The primary outcome variable of interest was traumatic exposures during migration, categorized as physical violence, sexual violence, violence against family or friends, violence by organized criminal groups, violence by government organizations, forced labor, and extortion or robbery (eTable in Supplement 1). Posttraumatic stress disorder was assessed based on either documentation by the forensic examiner stating the participant’s presentation was consistent with PTSD or a formal score on the PTSD Checklist for *DSM-5*, when available. The demographic data in our study were collected using predefined categories on the intake form, which specified ethnicity rather than race. Ethnicity, when reported, was classified according to the original source documentation and recorded by the researchers given their potential relevance to the outcomes of interest. The available response options included: Asian; African, Afro-Latino, or Afro-Caribbean; Hispanic or Latin American; Middle Eastern; White; Other, and Not stated. “Other” included options such as Indigenous, Pacific Islander, or other multiracial backgrounds not captured by the other categories. Sex was documented in applications and included in the data abstraction. Conversely, gender identity was reported in a minority of cases, typically when relevant to the asylum claim (eg, persecution based on gender identity), so it was not systematically included in analyses, although LGBTQ+ (lesbian, gay, bisexual, transgender, queer, questioning, and other) status, when reported, was recorded (Table 1). Data abstraction was performed by 3 researchers (K.J., S.M., and K.E.). Fifteen percent of cases were dual-abstracted to assess interrater reliability, showing high agreement between abstractors (pooled κ = 0.81). The remaining cases were divided evenly among the 3 researchers.

**Table 1.** Demographic Characteristics of Study Population

Characteristic	No. (%)			
	Overall (N = 94)	Pre-2019 (n = 26)	Post-2019 MPP (n = 22)	Post-2019 non-MPP (n = 46)
Age, mean (SD), y	33.1 (10.1)	35.7 (11.3)	30.6 (6.9)	32.9 (10.6)
Sex				
Female	38 (40)	9 (35)	7 (32)	22 (48)
Male	56 (60)	17 (65)	15 (68)	24 (52)
LGBTQ+ identity	10 (11)	1 (4)	2 (9)	7 (15)
Geographic region of origin				
Africa	20 (21)	9 (35)	3 (14)	8 (17)
Asia	3 (3)	2 (8)	0	1 (2)
Latin America	68 (72)	13 (50)	19 (86)	36 (78)
Other^a^	3 (3)	2 (8)	0	1 (2)
Reason for asylum claim^b^				
Nationality	2 (2)	1 (4)	1 (5)	0
Political opinion	29 (31)	9 (35)	8 (36)	12 (26)
Race and ethnicity	6 (6)	2 (8)	3 (14)	1 (2)
Religion	9 (10)	4 (15)	3 (14)	2 (4)
Social group	75 (80)	18 (69)	18 (81)	40 (87)
Other^c^	20 (21)	5 (19)	6 (27)	10 (22)

Abbreviations: LGBTQ+, lesbian, gay, bisexual, transgender, queer, questioning, and other; MPP, Migrant Protection Protocols.

^a^ Other regions include 1 patient from the Middle East and 2 patients from Russia.

^b^ Reasons for asylum not mutually exclusive.

^c^ Includes sexual assault, domestic violence, police violence, gang violence, and language-related persecution.

### Statistical Analysis

Statistical analysis was conducted from January 2023 to May 2025 using Stata, version 17 (StataCorp LLC). Logistic regression was performed to evaluate the association of exposure to the MPP with trauma during migration. Specifically, the odds of an asylum seeker reporting any of the traumatic events listed during migration was measured. To limit potential confounders with the year of evaluation and changing asylum landscape, we divided patients into 3 groups anchored on the introduction of the MPP in January 2019: asylum seekers from 2016 to 2018 (n = 26), asylum seekers from 2019 to 2022 exposed to the MPP (n = 22), and asylum seekers from 2019 to 2022 who were not exposed to the MPP due to exemptions or alternative entry points (n = 46). *Post-2019* is used to refer to the group that includes the years 2019 through 2022. Participants in the post-2019 non-MPP group were excluded from the MPP for a variety of reasons including alternate entry points and exemption from the policy based on home country.

Statistical tests were 2-sided. Logistic regression was performed between the pre-2019 group and both the post-2019 MPP and post-2019 non-MPP groups to calculate the odds that any traumatic event occurred during migration, reported as odds ratio (ORs) with 95% CIs and a preestablished statistical significance of α = .05. Additional sensitivity analysis was completed with only participants with a Latin American country of origin to assess for regional bias. Additional adjustments for other factors (age, sex, and reason for asylum) were not performed because of the small sample size.

Qualitative analysis was performed using a deductive approach. Quotations from affidavits and forensic examinations of MPP asylum seekers were reviewed and grouped into predefined trauma categories. Selected illustrative quotations were chosen to highlight key themes. All quotations were deidentified and pulled directly from source data. Some appear in first or third person, reflecting the way they were originally documented by attorneys or clinicians.

## Results

Among 94 participants, the mean (SD) age was 33.1 (10.1) years, 56 (60%) were men and 38 (40%) were women, and 68 (72%) originated from Latin America, with Africa the next most common region (20 [21%]) (Table 1). Additional participants were from Asia, the Middle East, and Russia. The most common asylum claim across all groups was persecution based on social group membership, reported by 75 participants (80%), followed by political opinion, reported by 29 participants (31%). The MPP group had a younger mean (SD) age of 30.6 (6.9) years and higher percentage of participants from Latin America (19 of 22 [86%]).

The primary outcome, trauma reported during migration, was higher in the MPP group (14 of 22 participants [64%]) compared with in the pre-2019 group (3 of 26 participants [12%]) (OR, 9.20; 95% CI, 2.12-39.89) (Table 2). The post-2019 group not exposed to the MPP did not have higher odds of trauma (OR, 1.38; 95% CI, 0.32-5.85). In a sensitivity analysis limited to participants from Latin America, the OR for trauma between the pre-2019 and MPP group was 4.40 (95% CI, 0.75-25.84), which did not reach statistical significance.

**Table 2.** Odds of Migration-Related Trauma and PTSD by Policy Exposure

Outcome	Pre-2019 (n = 26)	Post-2019 MPP (n = 22)	Post-2019 non-MPP (n = 46)	Odds ratio vs pre-2019 (95% CI)^a^
Reported trauma during migration, No. (%)	3 (12)	14 (64)	7 (15)	MPP: 9.20 (2.12-39.89); non-MPP: 1.38 (0.32-5.85)
PTSD, No. (%)	11 (42)	21 (95)	36 (7)	MPP: 28.64 (3.33-246.25); non-MPP: 4.91 (1.72-13.99)

Abbreviations: MPP, Migrant Protection Protocols; PTSD, posttraumatic stress disorder.

^a^ Odds ratios compare each post-2019 group with the pre-2019 reference group.

As a secondary outcome, the clinical examiner’s impression of PTSD was higher in the MPP group, (21 of 22 participants [95%]) than in the pre-2019 group (11 of 26 [42%]) (OR, 28.64; 95% CI, 3.33-246.25) (Table 2). In the post-2019 non-MPP group, 36 of 46 participants (78%) were assessed as having PTSD, a higher rate than the pre-2019 group (OR, 4.91; 95% CI, 1.72-13.99).

In addition to the quantitative patterns of trauma prevalence, we examined qualitative narratives to further characterize the nature of traumatic experiences. Thematic analysis of affidavits and forensic examinations highlighted different forms of trauma reported by asylum seekers. Major themes included physical and sexual violence, violence directed toward family or friends, police violence, gang violence, extortion, forced labor, and robbery. These narratives contextualize the quantitative findings by illustrating the lived experiences of asylum seekers. Representative deidentified quotes for each theme are presented in Table 3 and map onto the higher rate of migration-related trauma observed quantitatively in the MPP group.

**Table 3.** Quotations From MPP Asylum Seekers on Traumatic Experiences During Migration

Type of traumatic event	Deidentified quotations from affidavit or forensic medical examination^a^
Physical violence	They grabbed me and one of them scraped me down my chest with something sharp he had in his hand. They told me that they had found me and they were going to “finish me off.” I understood that this meant they intended to kill me… I worry all the time that they might have followed me home and might know where I’m staying.
Sexual violence	She discovered that an older child had molested her 3-year-old son. She was without any money or capacity to go see any medical professional regarding what might have happened to her son or to have therapy started if he had been affected by it. She feels although she fled from Venezuela to save his life and future, her inability to enter the US forcing them to stay with strangers and unable to choose safe lodging has led to this happening to her son.
Violence against family or friends	It has been difficult being in Mexico. We do not feel safe in Mexico. The neighbors told us that a child was stolen from the kindergarten near where we are living and they warned us not let our daughter be away from us.
Violence by organized criminal group	He has been robbed on 4 separate occasions since his arrival in Mexico. He has been threatened by gang members here, including menacing messages he has received on his cell phone (“I know who you are, what your name is, where you come from, where you live, where you walk, with whom you meet, when you go to the bathroom”).
Violence by government organization	We arrived the day US officials shot tear gas at the border. There was lots of chaos and harmed people at the shelter. The shelter was packed and living conditions were shocking. I questioned why I had come in the first place. I felt very disillusioned. I just wanted to be safe and protected.
Forced labor	I traveled on top of a train that was going toward a northern city in Mexico. A man came to me and said that he could help me cross to the other side if I helped him carrying a backpack. I was scared he would hurt or kill me if I refused. I only accepted to gain time to figure out how I could escape, because I knew what the consequences are of refusing the demands of narcotraffickers (torture or death).
Extortion or robbery	I have received threats from my landlord, who is a Mexican police officer. He is always armed, and he steals money from migrants when they leave the apartment. I stood up to him after he stole money from a Haitian residing in the building. The officer pulled me aside and threatened to harm me if I did not stop advocating for people.

Abbreviation: MPP, Migrant Protection Protocols.

^a^ Quotations are deidentified and appear in first or third person, depending on whether they were recorded as direct testimony or paraphrased by attorneys or clinicians in the original documentation.

## Discussion

This cohort study found that exposure to the MPP was associated with higher odds of migration-related trauma and PTSD among asylum seekers. These results highlight the association of MPP exposure with migration-related trauma and its potential downstream health outcomes. We discuss these findings in 3 areas: demographic, trauma and protective factors, and policy implications.

First, we examined demographic patterns across groups. Asylum seekers exposed to the MPP were the youngest group in our cohort and had the highest proportion from Latin America. This finding aligns with recent studies showing younger age distributions among asylum seekers in the US.^20^ Several factors may explain this finding. First, the MPP was originally designed to apply primarily to individuals from Latin American countries traversing Mexico, which shifted the affected population toward primarily Central American nationals. Second, trends from the Northern Triangle countries (Guatemala, Honduras, and El Salvador) in particular have shown an increase in younger migrants over the past decade.^12^ Data from the World Bank correlate with this finding, showing that Central American asylum seekers are increasingly arriving in their early 20s with a growing proportion of women and families compared with previous years.^21^ These demographic trends are consistent with our finding that the MPP group skewed younger than other groups in our cohort.

These results are consistent with previous research showing high rates of trauma at the US-Mexico border.^12,14,15,16,22,23^ Systemic gang violence was a common reason asylum seekers in our cohort fled their home country, and reports document that gang-related murders of deported asylum seekers and continued exploitation during migration continue to be problems facing migrants in Mexico.^22^ Returned migrants, known as “retornados,” face marginalization and risk revictimization.^16^ In a Mexican migrant encampment study, women expressed widespread fear of sexual violence and distrust of authorities.^23^ Such themes are demonstrated in our forensic evaluations (Table 3). For example, 1 participant described repeated robberies and direct threats from gang members, including receiving messages stating “I know where you are… where you live.” Another woman described an inability to secure safe lodging in Mexico, which led to sexual violence against her child.

In contrast, asylum seekers after 2019 who were not subjected to the MPP did not report higher rates of trauma. Access to safer communities and services in the US may have been protective against trauma. Research suggests that protective factors such as social support, faith, and civic engagement have been associated with decreased migrant stress.^24^ Those admitted to the US likely benefited more from these factors. However, while protective factors may mitigate the association of migration-related trauma, the more important distinction is that individuals exposed to the MPP were more likely to be placed in high-risk environments such as tent cities or unsafe border regions with inherently higher trauma exposure.

Similar patterns have emerged globally, where restrictive policies and high-risk environments have been associated with higher rates of trauma and a greater burden of mental health disease among displaced populations. Research on Syrian refugees living in tent-city camps documented overcrowding, insecurity, and limited access to basic services being associated with a high rate of PTSD.^25^ Médecins Sans Frontières reported that among a group of migrants who crossed the Mediterranean to Italy, over 80% of respondents experienced torture either in their country of origin or in transit, most commonly at the hands of traffickers (61%) or law enforcement (29%).^26^ More than one-third of these incidents occurred in countries designated by European authorities as “safe” for migrants. This parallels the experiences of individuals in our cohort who, despite being returned to Mexico under the MPP, continued to report unsafe and harmful conditions. Similarly, a study of nearly 500 Rohingya refugees in Bangladesh found high rates of depression and PTSD that were linked not only with exposure to previous trauma but also ongoing daily stressors such as overcrowding, food insecurity, and violence and crime in their refugee camps.^27^ Together, these findings highlight how restrictive migration and asylum policies such as the MPP that compel migrants to remain in unsafe environments can perpetuate rather than alleviate trauma among displaced populations.

These results should be interpreted in the broader context of ongoing US border policy debates. Although the MPP aimed to manage migration and strengthen border security, it was associated in our study with higher reported rates of violence, exploitation, and psychological distress. This finding adds to evidence that deterrent-based approaches may be associated with higher rates of migrant trauma and reliance on riskier migration routes, compounding the premigration trauma that compelled many asylum seekers to flee their home countries in the first place.^28^

Despite its suspension in 2021, the MPP was reinstated in January 2025 and continues to shape the asylum process.^7^ Other recent measures, such as the US Department of Homeland Security Third Country Removals,^29^ permit deportation to countries where assurances of migrant safety are uncertain. Although such policies may reduce cross-border entry or perpetuate the image of security in the short term, our results suggest that they may do so at the cost of exposing migrants to unsafe environments. Policymakers should therefore weigh not only border security objectives but also the public health and human rights consequences of these policies.

These findings highlight the importance of considering the health and safety of asylum seekers in the design of asylum policies. Potential approaches such as expanded legal pathways, increased access to mental and trauma support, and targeted international initiatives to address root causes of migration, such as systemic violence and economic instability in countries of origin, could reduce the harms observed in this study. If the MPP continues, a more robust multinational effort to ensure asylum seekers receive adequate protection and basic social support would be important for their health and safety.

### Limitations

This study has several limitations. First, the sample was drawn from a medicolegal clinic that evaluates primarily asylum seekers who have obtained legal representation and undergone forensic medical examinations. Amnesty International has estimated that only 5% of asylum seekers subject to the MPP have been able to obtain legal representation, so our patient population likely represents a subgroup with greater access to legal and medical resources.^6^ Second, the retrospective design of this study limits causal inference and relies on preexisting documentation, which may vary in documentation style and introduce reporting bias. For example, the higher rate of PTSD observed in both the MPP and post-2019 non-MPP groups may reflect increased attention to screening and documentation of PTSD in recent years rather than true differences among groups.

Preexisting conditions and socioeconomic factors were not controlled for and may have confounded the observed associations. The MPP group skewed younger and had a larger proportion of migrants from Central America. A planned sensitivity analysis limited to Latin American participants showed that the odds of migration-related trauma in the MPP group was not significantly different compared with the pre-2019 group, highlighting that migration from Latin America, a region heavily affected by violence and instability, may be an independent risk factor in itself for migration-related trauma.^12^ In addition, logistic regression analyses were unadjusted given the small sample size, which limited our ability to control for all factors. Finally, other asylum policies implemented after 2019, such as the asylum transit ban and changes to US Customs and Border Protection processing, may have contributed to the observed differences, making it difficult to isolate the associations of MPP alone.

## Conclusions

In this retrospective cohort study of asylum seekers before and after the introduction of the MPP in 2019, exposure to the MPP was associated with higher rates of trauma experiences during migration and a higher rate of PTSD reported in forensic medical examinations. These findings highlight the possible health consequences of restrictive border policies and underscore the need for immigration policies that consider the safety and well-being of asylum seekers throughout the migration process.

## Notes

Supplement 1. **eFigure.** Flow Diagram of Participant Selection **eTable.** Subtypes of Trauma Reported by Policy Exposure

Supplement 2.
Data Sharing Statement

## References

[1] Who we protect: asylum-seekers. The UN Refugee Agency. Accessed September 5, 2025. https://www.unhcr.org/us/about-unhcr/who-we-protect/asylum-seekers

[2] Data and statistics: global trends. The UN Refugee Agency. June 12, 2025. Accessed September 5, 2025. https://www.unhcr.org/us/global-trends

[3] Immigration court quick facts. Transactional Records Access Clearinghouse (TRAC). Accessed September 5, 2025. https://tracreports.org/immigration/quickfacts/eoir.html

[4] The “Migrant Protection Protocols”: an explanation of the Remain in Mexico Program. American Immigration Council. February 1, 2024. Accessed August 26, 2025. https://www.americanimmigrationcouncil.org/fact-sheet/migrant-protection-protocols/

[5] Remain in Mexico: unlawful, ineffective, and must never return. Human Rights First. January 10, 2025. Accessed September 5, 2025. https://humanrightsfirst.org/library/remain-in-mexico-unlawful-and-ineffective/

[6] Anniversary of the implementation of so-called “Remain in Mexico” policy marks rampant rights abuses. Amnesty International USA. January 24, 2020. Accessed September 5, 2025. https://www.amnestyusa.org/press-releases/anniversary-of-the-implementation-of-so-called-remain-in-mexico-policy-marks-rampant-rights-abuses/

[7] DHS reinstates Migrant Protection Protocols, allowing officials to return applicants to neighboring countries. US Department of Homeland Security. January 21, 2025. Accessed September 5, 2025. https://www.dhs.gov/news/2025/01/21/dhs-reinstates-migrant-protection-protocols

[8] Circumvention of lawful pathways. *Federal Register*. May 16, 2023. Accessed September 5, 2025. https://www.federalregister.gov/documents/2023/05/16/2023-10146/circumvention-of-lawful-pathways

[9] Control of communicable diseases; foreign quarantine: suspension of introduction of persons into United States from designated foreign countries or places for public health purposes. *Federal Register*. March 24, 2020. Accessed September 5, 2025. https://www.federalregister.gov/documents/2020/03/24/2020-06238/control-of-communicable-diseases-foreign-quarantine-suspension-of-introduction-of-persons-into

[10] Blackmore R, Boyle JA, Fazel M, . The prevalence of mental illness in refugees and asylum seekers: a systematic review and meta-analysis. PLoS Med. 2020;17(9):e1003337. doi:10.1371/journal.pmed.1003337 32956381 PMC7505461

[11] Steel Z, Chey T, Silove D, Marnane C, Bryant RA, van Ommeren M. Association of torture and other potentially traumatic events with mental health outcomes among populations exposed to mass conflict and displacement: a systematic review and meta-analysis. JAMA. 2009;302(5):537-549. doi:10.1001/jama.2009.1132 19654388

[12] Cuneo CN, Huselton KE, Praschan NC, Saadi A, Gartland MG. What counts as “safe?”: exposure to trauma and violence among asylum seekers from the Northern Triangle. Health Aff (Millwood). 2021;40(7):1135-1144. doi:10.1377/hlthaff.2021.00082 34228513

[13] Gostin LO, Friedman EA. Title 42 exclusions of asylum seekers—a misuse of public health powers. JAMA Health Forum. 2023;4(1):e230078. doi:10.1001/jamahealthforum.2023.0078 36656601

[14] Fabi R, Rivas SD, Griffin M. Not in our name: the disingenuous use of “public health” as justification for Title 42 expulsions in the era of the Migrant Protection Protocols. Am J Public Health. 2022;112(8):1115-1119. doi:10.2105/AJPH.2022.306887 35737927 PMC9342824

[15] Neusner J, Kizuka K. The nightmare continues: Title 42 court order prolongs human rights abuses, extends disorder at U.S. borders. Human Rights First. June 16, 2022. Accessed August 26, 2025. https://humanrightsfirst.org/library/the-nightmare-continues-title-42-court-order-prolongs-human-rights-abuses-extends-disorder-at-u-s-borders/

[16] Mercado A, Venta A, Morales F, . Trauma in the American asylum process: experiences of immigrant families under the Migrant Protection Protocols. Psychol Trauma. 2024;16(suppl 2):S379-S388. doi:10.1037/tra0001368 36222662 PMC10083184

[17] Silverstein MC, Long RFP, Burner E, Parmar P, Schneberk TW. Continued trauma: a thematic analysis of the asylum-seeking experience under the Migrant Protection Protocols. Health Equity. 2021;5(1):277-287. doi:10.1089/heq.2020.0144 34095707 PMC8175263

[18] Atkinson HG, Wyka K, Hampton K, . Impact of forensic medical evaluations on immigration relief grant rates and correlates of outcomes in the United States. J Forensic Leg Med. 2021;84:102272. doi:10.1016/j.jflm.2021.102272 34743036

[19] Iacopino V, Haar RJ, Heisler M, . Istanbul Protocol 2022 empowers health professionals to end torture. Lancet. 2022;400(10347):143-145. doi:10.1016/S0140-6736(22)00948-5 35779555

[20] Newton-Dame R, Jacobson L, Wallach AB, Silverman E, Dreyer B, Long T. Caring for patients seeking asylum: early data from the safety net system in New York City. J Public Health Manag Pract. 2025;31(3):477-485. doi:10.1097/PHH.0000000000002106 39724081 PMC12316154

[21] Mejia-Mantilla C, González Rubio S. A journey through borders: understanding migration in Central America. World Bank Blogs. January 18, 2024. Accessed September 4, 2025. https://blogs.worldbank.org/en/latinamerica/understanding-migration-central-america

[22] De Jesús-Rentas G, Boehnlein J, Sparr L. Central American victims of gang violence as asylum seekers: the role of the forensic expert. J Am Acad Psychiatry Law. 2010;38(4):490-498. 21156907

[23] Reynolds CW, Ramanathan V, Das PJ, Schmitzberger FF, Heisler M. Public health challenges and barriers to health care access for asylum seekers at the U.S.-Mexico border in Matamoros, Mexico. J Health Care Poor Underserved. 2022;33(3):1519-1542. doi:10.1353/hpu.2022.0127 36245178

[24] Arce MA, Kumar JL, Kuperminc GP, Roche KM. “*Tenemos que ser la voz*”: exploring resilience among Latina/o immigrant families in the context of restrictive immigration policies and practices. Int J Intercult Relat. 2020;79:106-120. doi:10.1016/j.ijintrel.2020.08.006 32943805 PMC7491871

[25] Cratsley K, Brooks MA, Mackey TK. Refugee mental health, global health policy, and the Syrian crisis. Front Public Health. 2021;9:676000. doi:10.3389/fpubh.2021.676000 34414156 PMC8369241

[26] Inhuman: torture Along the Mediterranean migration route, and the support of survivors in a fragile system. Médecins Sans Frontières. June 2025. Accessed June 26, 2025. https://msf.org.pt/wp-content/uploads/sites/4/2025/06/MSF-report_Inhuman.-Torture-along-the-Mediterranean-migration-route-and-the-support-of-survivors-in-a-fragile-system.pdf

[27] Riley A, Akther Y, Noor M, Ali R, Welton-Mitchell C. Systematic human rights violations, traumatic events, daily stressors and mental health of Rohingya refugees in Bangladesh. Confl Health. 2020;14(1):60. doi:10.1186/s13031-020-00306-9 32843894 PMC7441657

[28] Tenorio A, Hill LL, Doucet JJ. A border health crisis at the United States–Mexico border: an urgent call to action. Lancet Reg Health Am. 2024;31:100676. doi:10.1016/j.lana.2024.100676 38304757 PMC10827580

[29] Metzler J. What are third-country deportations, and why is Trump using them? Council on Foreign Relations. September 3, 2025. Accessed November 10, 2025. https://www.cfr.org/article/what-are-third-country-deportations-and-why-trump-using-them

