# Supplementary material for: Migration-Related Trauma Among Asylum Seekers Exposed to the Migrant Protection Protocols
Source: JAMA Netw Open. 2026 Jan 6;9(1):e2550786. doi: 10.1001/jamanetworkopen.2025.50786 (PMC12776200; doi:10.1001/jamanetworkopen.2025.50786)
Supplement: Supplement 1. — eFigure. Flow Diagram of Participant Selection eTable. Subtypes of Trauma Reported by Policy Exposure [file jamanetwopen-e2550786-s001.pdf]

Supplementary Online Content

Joyner K, Burner E, Axeen S, Murugan S, English K, Schneberk TW. Migration-related trauma among asylum seekers exposed to the Migrant Protection Protocols. *JAMA Netw Open.* 2025;8(12):e2550786.
doi:10.1001/jamanetworkopen.2025.50786

eFigure. Flow Diagram of Participant Selection

eTable. Subtypes of Trauma Reported by Policy Exposure

This supplementary material has been provided by the authors to give readers additional information about their work.

© 2025 Joyner K et al. *JAMA Network Open.*

eFigure. Flow Diagram of Participant Selection

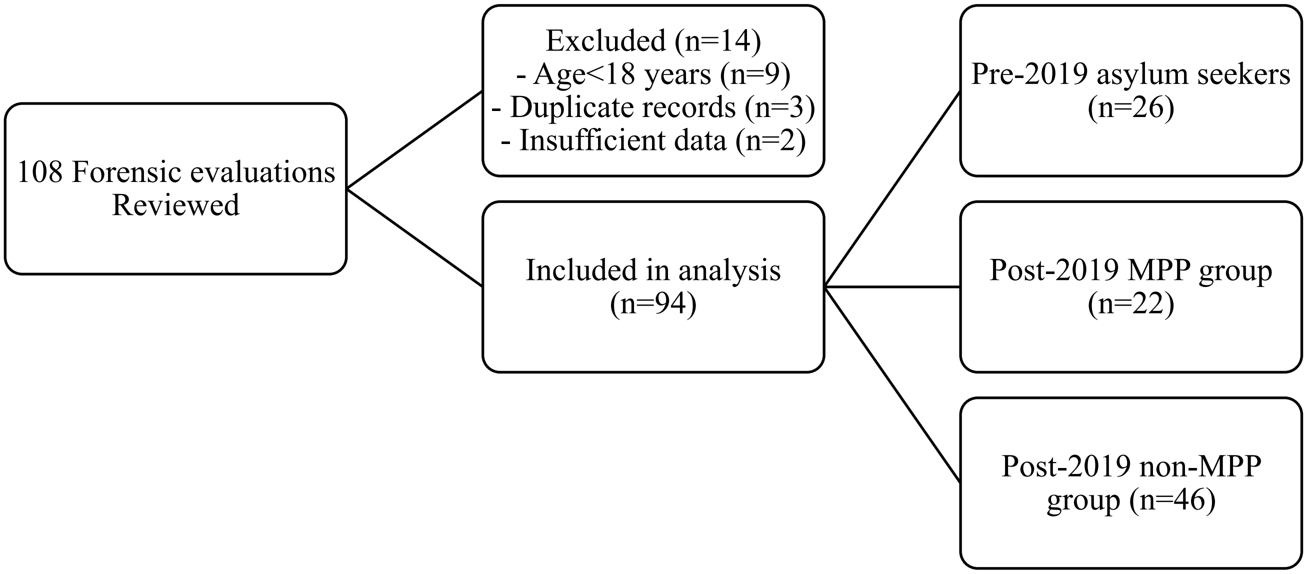

© 2025 Joyner K et al. *JAMA Network Open.*

eTable. Subtypes of Trauma Reported by Policy Exposure

Traumatic Event	Location	Pre-2019, No. (%) (n=26)	Post-2019 MPP, No. (%) (n=22)	Post-2019 non-MPP, No. (%) (n=46)
**Physical violence**	Home country	25 (96%)	21 (95%)	44 (96%)
	Migration	*3 (12%)*	*6 (27%)*	*1 (2%)*
**Sexual violence**	Home country	6 (23%)	9 (41%)	22 (48%)
	Migration	*0 (0%)*	*2 (9%)*	*2 (4%)*
**Violence against family/friends**	Home country	14 (54%)	20 (91%)	27 (59%)
	Migration	*0 (0%)*	*2 (9%)*	*0 (0%)*
**Violence by organized criminal group**	Home country	9 (35%)	13 (59%)	26 (57%)
	Migration	*0 (0%)*	*4 (18%)*	*2 (4%)*
**Violence by government organization**	Home country	13 (50%)	9 (41%)	15 (33%)
	Migration	*0 (0%)*	*2 (9%)*	*1 (2%)*
**Forced labor**	Home country	4 (15%)	3 (14%)	5 (11%)
	Migration	*0 (0%)*	*2 (9%)*	*1 (2%)*
**Extortion or robbery**	Home country	6 (23%)	6 (27%)	15 (33%)
	Migration	*1 (4%)*	*8 (36%)*	*3 (7%)*

© 2025 Joyner K et al. *JAMA Network Open.*
