# Supplementary material for: Migration-Related Trauma Among Asylum Seekers Exposed to the Migrant Protection Protocols
Source: JAMA Netw Open. 2026 Jan 6;9(1):e2550786. doi: 10.1001/jamanetworkopen.2025.50786 (PMC12776200; doi:10.1001/jamanetworkopen.2025.50786)
Supplement: Supplement 2. — Data Sharing Statement [file jamanetwopen-e2550786-s002.pdf]

Data Sharing Statement

Joyner. Migration-Related Trauma Among Asylum Seekers Exposed to the Migrant Protection Protocols. *JAMA Netw Open*. Published December 22, 2025.
doi:10.1001/jamanetworkopen.2025.50786

Data
Data available: No

Additional Information
Explanation for why data not available: sensitive data regarding refugees
